# A Review of Myeloablative vs Reduced Intensity/Non-Myeloablative Regimens in Allogeneic Hematopoietic Stem Cell Transplantations

**DOI:** 10.4274/balkanmedj.2017.0055

**Published:** 2017-01-05

**Authors:** Erden Atilla, Pınar Ataca Atilla, Taner Demirer

**Affiliations:** 1 Department of Hematology, Ankara University School of Medicine, Ankara, Turkey

**Keywords:** Conditioning regimens, myeloablative, non-myeloablative, reduced-intensity

## Abstract

Allogeneic hematopoietic stem cell transplantation (Allo-HSCT) is a curative treatment option for both malignant and some benign hematological diseases. During the last decade, many of the newer high-dose regimens in different intensity have been developed specifically for patients with hematologic malignancies and solid tumors. Today there are three main approaches used prior to allogeneic transplantation: Myeloablative (MA), Reduced Intensity Conditioning (RIC) and Non-MA (NMA) regimens. MA regimens cause irreversible cytopenia and there is a requirement for stem cell support. Patients who receive NMA regimen have minimal cytopenia and this type of regimen can be given without stem cell support. RIC regimens do not fit the criteria of MA and NMA: the cytopenia is reversible and the stem cell support is necessary. NMA/RIC for Allo-HSCT has opened a new era for treating elderly patients and those with comorbidities. The RIC conditioning was used for 40% of all Allo-HSCT and this trend continue to increase. In this paper, we will review these regimens in the setting of especially allogeneic HSCT and our aim is to describe the history, features and impact of these conditioning regimens on specific diseases.

## INTRODUCTION

Treatment regimen used for hematopoietic stem cell transplantation (HSCT) must accomplish two goals depending on the patient’s disease and the source of stem cells. Since the majority of allogeneic transplantations are performed for the treatment of malignant disease, the regimen must provide tumor cytoreduction and ideally disease eradication (1,2,3,4,5,6,7,8). In the case of allogeneic HSCT the regimen must be sufficiently immunsuppressive to overcome host rejections of the donor stem cells. Most of the high dose chemotherapy regimens defined have been utilized in patients with hematological malignancies in the setting of allogeneic transplantations (9,10,11,12,13,14,15,16,17,18).

Several studies showing high doses of total body irridation (TBI) causes death from marrow failure and intravenous infusion of marrow or spleen cells after TBI prevents death in mice attracted many researchers to investigate human transplantations in mid-1950s (19). Early trials of human marrow grafting had been unsuccessful since patients failed to engraft or developed fatal graft-versus-host disease (GVHD) (20). In late 1960’s, investigators had detected that dog leukocyte antigen compatibility between donors and recipients as well as effective drugs to overcome GVHD improved the outcome of Allo-HSCT (21,22). Thomas et al. (23) had reported the results of 100 patients with various hematologic malignancies and aplastic anemia with long-term disease-free survival (DFS) despite high transplant related mortality (TRM) in 1975. Starting from second half of the 1960s up to 1980s cyclophosphamide and/or TBI as high-dose conditioning regimens were preferred. Busulfan, another alkylating agent, was started to be used as an alternative agent to TBI based regimens and combined with cyclophosphamide in 1983 (24).

Conventional ablative allo-HSCT depends on the tolerated doses of systemic chemo-radiotherapy in order to eradicate malignant tumor burden. This resulted with high regimen-related toxicities in elderly and patients with comorbidities. In considering that, most hematological diseases occur at ages from 65 to 70 (25), investigators started to seek for less ablative and less toxic conditioning regimens for special populations such as elderly and patients with comorbidities in 1990s. Several studies documented that reduced intensity preparative regimen followed by stem cell infusion was associated with mixed chimerism and then full chimerism with a documented graft vs leukemia (GVL) effect in the setting of hematologic malignancies and graft versus tumor (GVT) effect in the setting of solid tumors (26,27,28,29,30,31,32). GVL or GVT effect can be supported by higher risk of relapse in patients who do not develop GVHD, who receives T-cell depleted grafts or those receiving grafts from identical twins and documentation of high remission rates after donor lymphocyte infusions (33). In addition to donor natural killer and B cells, the recognition of host-specific minor or major histocompatibility antigens by donor T cells may be the mechanisms of GVT effects (34,35).

The MA/NMA/RIC conditioning regimens currently in use summarized in Table 1 (36). Most known RIC regimens include fludarabine and intermediate doses of alkylating agents such as thiotepa, melphalan and busulfan whereas NMA regimens usually contain low-dose (2 Gy) total body irradiation (TBI) with or without fludarabine (37). Such conditioning regimens may cause mild myelosuppression, low treatment-related toxicity and antitumor responses which may continue for extended periods of time. The known complications of Allo-HSCT such as pancytopenia, mucositis and organ damage occur less frequently with RIC regimens. It has been suggested that RIC regimens might be associated with an improved survival and lower incidence of relapse than NMA conditioning (38). However Mohty et al. (39) compared the outcomes of NMA (n=323) vs RIC regimens (n=877) in acute myeloid leukemia (AML) and found similar two-year DFS rates between two groups (50% vs 53%, respectively) (39).

It is well known that a complete donor T-cell chimerism is correlated with a low risk of relapse or progression. MA regimens leads to state of full chimerism rather earlier than RIC regimens. Mixed chimerism is detected initially after transplant in most RIC conditioning regimens and in fact graft rejection is more common in RIC Allo-HSCT patients compared to conventional regimens (40). Myeloablative regimens are usually associated with higher incidence of acute GVHD but a similar incidence of chronic GVHD compared to RIC regimens (41). The risk of pancytopenia following RIC conditioning is associated with a lower risk of bacteremia but the risk of fungal infections are rather similar after MA or RIC conditioning (42). The incidences of cytomegalovirus reactivation and disease as well as BK virus-associated hemorrhagic cystitis were not found to be different in MA and RIC regimens (43) but decreased mucosal and early-late organ toxicities occur after RIC regimens (44). Studies in immune reconstitution following allo-HSCT have demonstrated various results since the use of different RIC protocols in different spectrum and some authors have shown a rapid recovery of total lymphocytes, Tregs and memory and naive CD4+ lymphocytes after RIC regimens (45).

### Reduced Intensity Conditioning vs Myeloablative Conditioning in Specific Diseases

### Acute Leukemias

In AML, several retrospective comparisons of RIC and MA conditioning regimens are difficult to evaluate due to different patient populations. Patients who received RIC regimens had more high risk features and comorbidities than those received MA conditioning but the survival rates were found to be similar between RIC and MA conditioning (46). Several comparative studies of RIC vs MA in AML is shown in Table 2. The largest retrospective trial was reported by Center for International Blood and Marrow Transplant Research (CIBMTR) in which higher relapse and inferior survival rates were detected after RIC transplants (47). In a subgroup analysis, the better DFS was shown in patients with favorable characteristics (good performance status, age 40-60, AML in CR1). Bornhauser et al. (48) published a prospective randomized study comparing flu/TBI with Cy/TBI and there were no significant difference in rates of DFS, overall survival (OS), non-relapse mortality (NRM) or relapse. Younger ages of patients and more intense RIC regimen than regular were negative drawbacks of the study. Sebert et al. (49) compared the MA with RIC in patients aged 35 years and over in AML and found that relapse rates and OS were similar. After adjusting for gender, donor/recipient mismatch, cytogenetic risk and CD34+ cells, NRM was significantly lower with the RIC regimen (p=0.027). Therefore, generally today RIC regimens might be a good choice for patients with AML who have significant comorbidities or older age (50).

Increased NRM in elderly patients with comorbidities may worsen the outcome of MA allo-HSCT in acute lymphoblastic leukemia (ALL). Marks et al. (51) had not detected any impact of the conditioning intensity on relapse risk or transplant-related mortality (TRM) in Ph negative ALL patients in first or second complete remission (CR) who received allografts from siblings or unrelated donors, (51). Mohty et al. (52) from EMBT group could not demonstrate any effect of conditioning regimen in 127 RIC allo-HSCT and 449 MA related allo-HSCT transplants on leukemia-free survival who were in first or second CR. Hematopoietic Cell Transplantation Society of Japan analyzed 369 MA vs 206 RIC allo-HSCT and there were no statistically significant differences in 3-year OS, DFS and NRM: 51% vs 53%, 47% vs 39% and 38% vs 36%, respectively. RIC regimens were associated with a better OS and DFS in patients who received HLA-mismatched transplantation and were aged ≥55 years (Table 2) (53). Based on these controversial literature data regarding the comparison of RIC vs myeloablative conditioning (MAC) in patients with ALL, one may conclude that further prospective trials and head to head comparisons are needed in order to evaluate efficacy of RIC or MAC regimens in ALL.

Recently, the results of phase III multi-center randomized study of Blood and Marrow Transplant Clinical Trials Network (BMT CTN) 0901 have been released. This study compared outcomes on the basis of conditioning intensity in patients with Myelodysplastic syndrome (MDS) and AML. The study concluded that RIC results in higher relapse rates and lower TRM compared to MAC with a statistically significant advantage in RFS for patients receiving MAC. It has to be noted that inclusion of heterogeneous regimens in both RIC and MAC arms and lack of longer follow-up establishes the main weaknesses of the BMT CTN 0901 study (54).

### Myelodysplastic Syndrome, Myeloproliferative Neoplasms and Aplastic Anemias

There are some controversies regarding the impact of conditioning intensity on disease control in MDS. Warlick et al. (55) reported improved disease control with MA regimens. High risk MDS patients (n=43) were treated with MA and T cell depleted alloHSCT resulted in EFS at 1 and 3 years as 47% and 34%, respectively. The overall toxicity was detected to be similar compared to multiple recorded series using RIC regimens. Martino et al. (56) reported the results of 25 EBMT-affiliated centers including 215 patients. In that study 3-year incidences of relapse, NRM and OS were 45%, 22% and 41%, respectively, associated with the increased risk of TRM with MA regimen. Scott et al. (57) showed, in 150 patients with MDS or AML transformed from MDS, that NMA compared to MA regimen had no impact on three-year OS (27% vs 48%, p=0.56), progression free survival (PFS) (28% vs 44%, p=0.6) and NRM (41% vs 34%). Authors reported that there was no correlation between relapse rates and pre-transplant disease control in patients with MDS in contrast to the study showing that patients receiving MA conditioning had lower risk of relapse particularly those in CR (58).

Myeloproliferative neoplasms (MPNs) include a group of clonal and chronic hematologic disorders with similar features. Classical MPNs are chronic myeloid leukemia (CML), idiopathic/primary myelofibrosis (MF), polycythemia vera (PV), essential thrombocytopenia (ET), systemic mastocytosis, chronic neutrophilic leukemia and chronic eosinophilic leukemia (59). Today, patients with CML progressed to accelerated or blast phase or failed to second-generation tyrosine kinase inhibitors (TKIs) or have resistant mutations to TKI may undergo allo-HSCT. An earlier retrospective study by European Group for Blood and Marrow Transplantation (EBMT) Chronic Leukemia Working Party (CLWP) showed a reduction in the TRM with RIC regimens however this did not translate into a significantly improved 3-year survival in patients with EBMT scores of 0 to 2 (60). RIC regimens were usually preferred with cellular immunotherapy and TKIs in which TKIs act concurrently with donor lymphocyte infusion (61). Patients with MF in general are usually older and have comorbidities at the time of of allo-HSCT. In a retrospective cohort comparing MA and RIC regimens in 51 patients with MF observed no significant differences in 3-year OS or PFS (OS 44% vs 31%, PFS 44% vs 24%, respectively) however the relapse rate was lower 12% vs 46% in RIC with a strong trend toward significance (p=0.06) (62). In a prospective trial conducted by EBMT CLWP, there was no significant difference in NRM in between matched unrelated and HLA-matched sibling RIC transplants, 13% vs 10%, respectively. The conditioning regimen included busulfan, fludarabine and anti-thymocyte globulin (ATG) and 5-year relapse rate, PFS and OS were 20%, 51% and 67%, respectively (63). On the other hand, a recent study in 53 patients with MF demonstrated that the cumulative incidence of graft failure within 60 days after allo-HSCT was high (28%) and associated with the decreasing intensity of the conditioning regimen. Therefore, researchers have recommended to use more intensive conditioning regimens in MF (64). In a large retrospective CIBMTR analysis, 5 year-TRM in 117 patients with advanced PV and ET who received MA (n=80) and RIC/NMA (n=37) conditioning regimens was higher in MA group (40% vs 18%, p<0.05) as well as 1-year and 5-year relapse rates were lower in patients receiving MA regimens, 8% vs 33% (p=0.003) and 9% vs 41% (p<0.05), respectively. Last but not least 5-year survival rates were similar in both conditioning regimens (65). In considering the similar OS rates, older ages and comorbidities of these patients with PV and ET, RIC regimens can be more appealing strategy when it comes to allo-HSCT.

In newly diagnosed severe acquired idiopatic aplastic anemia patients younger than 30-40 years, allo-HSCT is the first line treatment of choice (66). The standart conditioning regimen for HLA identical sibling HSCT relies on cyclophophamide combined with or without ATG. However, for older patients the long term survival after HSCT was detected to be lower in both Seattle (67) and European cohorts (68). EBMT Severe Aplastic Anemia Working Party conducted a study comparing reduced intensity, fludarabine-low dose cytarabine and ATG, conditioning regimen versus standart myeloablative regimen in older aplastic anemia patients. The patients who received RIC (n=30) had higher probability of OS than the control group (n=239) when adjusting for recipient’s age (p=0.04). The acute and chronic GVHD incidence was detected to be similar in both groups. The authors concluded that RIC regimen might reduce the negative impact of age in older patients (69).

### Lymphomas and Chronic Lymphocytic Leukemia

In patients with Hodgkin’s lymphoma (HL), allo-HSCT is generally performed in relapsed disease after autologous HSCT or refractory disease status but MAC followed by allo-HSCT with conventional preparative regimens had been associated with high toxicity rates and TRM in this group of patients. In the era of reduced intensity regimens, EBMT Lymhoma Working Party reported a lower NRM and improved OS with RIC allo-HSCT in patients with relapsed or refractory HL (70). In a retrospective analysis, 285 patients with HL underwent RIC allo-HSCT without any risk factor and had a 3-year PFS and OS of 42% and 56% compared to 8% and 25% for patients with one or more risk factors. NRM was detected to be associated with age > 45, poor performance status, chemo-refractory disease and transplantation before 2002 (71). Sarina et al. (72) in a retrospective evaluation in 185 patients concluded that patients with HL relapsing after autologous HSCT have a survival advantage if they undergo RIC allo-HSCT.

Several studies have evaluated the efficacy HSCT following RIC or NMA conditioning as a treatment option in relapse or refractory non-HL (NHL) after failure of autologous HSCT. The PFS rate at 3-year was 82% in 18 refractory mantle cell lymphoma (MCL) patients treated with NMA conditioning containing fludarabine, cyclophosphamide and rituximab (73). Maris et al. (74) reported an OS and DFS of 65% and 60% in 33 relapsed-refractory MCL with 2 Gy of TBI and fludarabine. Whereas, no differences were seen regarding OS and PFS rates between RIC and MA regimens in the EBMT registry in patients with diffuse large B cell NHL relapsing after an autologous HSCT (75).

CLL has an indolent and prolonged clinical course and mostly patients are in older age group. Sorror et al. (76) reported 64 refractory CLL patients with median age of 56 underwent allo-HSCT with NMA conditioning. The 2-year estimated OS and DFS rates were 60% and 52% with the incidence of acute GVHD of grade II-IV of 61%. In a different study again, Sorror et al. (77) reported that the outcomes of 152 patients with refractory CLL (n=40) or lymphoma (n=112) who received NMA regimen were associated with a 3-year NRM and OS rates of 25% and 53%, respectively.

### Solid Tumors

Based on the growing knowledge on the immune system and T cell biology, allogeneic HSCT also represents a promising approach in some solid tumors. Several EMBT phase I and II studies which were conducted by Solid Tumors Working Party documented the presence of a graft-versus solid tumor effect in patients with various solid tumors such as renal, ovarian, colon and soft tissue sarcomas [27-78]. Aglietta et al. (30) reported the results of RIC allo-HSCT in 39 metastatic colorectal canter patients. Eighty percent of patients were in progressive disease at transplant and acute GVHD occurred in 35% of patients. Disease was partly controlled in 46% of patients. In a study reported by Thiel et al. (79) 30 patients with advanced rhabdomyosarcoma underwent allo-HSCT and 3-year OS was shown to be 20% associated with a cumulative risk of progression of 67%. Demirer et al. reported that tumor response in solid tumors was associated with the development of acute and chronic GVHD (72). Based on this, Demirer et al. (31) conluded that further improvements would depend on the identification of the antigen targets of GVT and reduction of the toxicity of the procedure. It is very reasonable to state that targeted therapies may improve the immune effect of allogeneic transplantation in a positive way in patient with solid tumors (32).

In conclusion, allogeneic stem cell transplantation is a curative approach in many diseases. The advantages of RIC regimens are lower toxicity profiles and lower NRM rates. RIC or NMA allo-HSCT can be a feasible option in geriatric patients and patients with comorbidities. Future studies are needed for a clear-cut understanding of the mechanisms of GVL and GVT effects of donor T cells and its subsets in order to optimize the efficacy of such treatment modalities as RIC or MAC in allo-HSCT (5).

## Figures and Tables

**Table 1:**
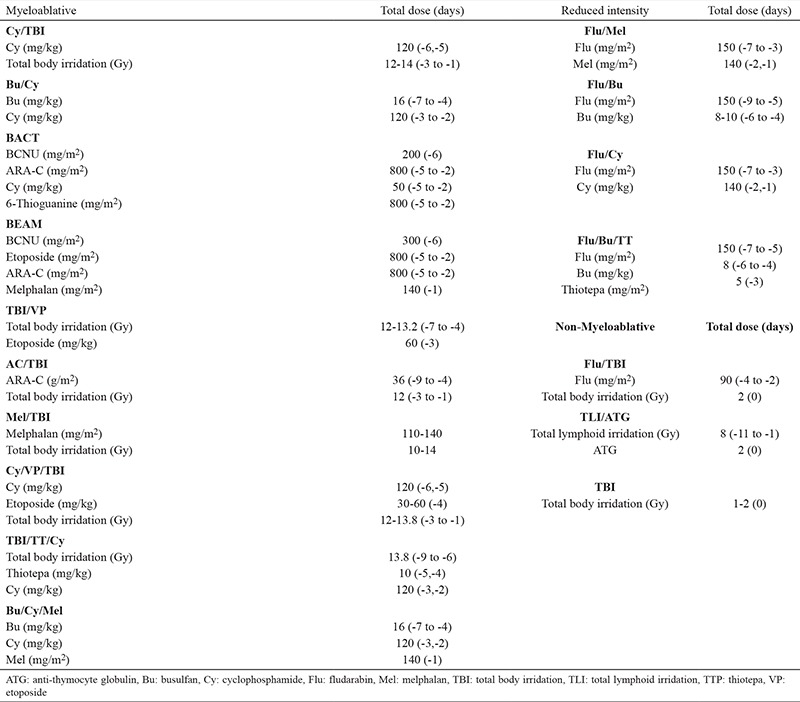
MA/NMA/RIC conditioning regimens currently in use

**Table 2:**
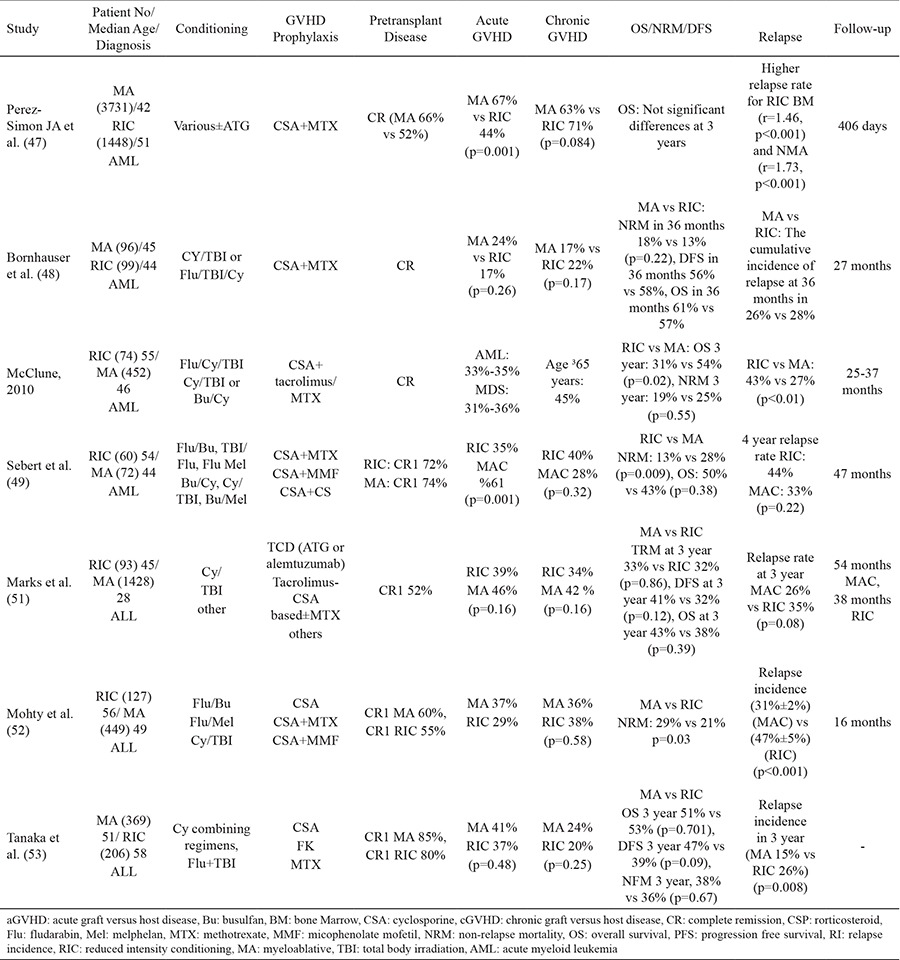
MA vs RIC regimen in acute leukemias
